# Using *S. cerevisiae* as a Model System to Investigate *V. cholerae* VopX-Host Cell Protein Interactions and Phenotypes

**DOI:** 10.3390/toxins7104099

**Published:** 2015-10-14

**Authors:** Christopher H. Seward, Alexander Manzella, Ashfaqul Alam, J. Scott Butler, Michelle Dziejman

**Affiliations:** 1 Carl R. Woese Institute for Genomic Biology, University of Illinois at Urbana-Champaign, Urbana, IL 61801, USA; E-Mail: seward2@illinois.edu; 2 Department of Microbiology and Immunology, University of Rochester School of Medicine & Dentistry, Rochester, NY 14642, USA; E-Mails: amanzel2@gmail.com (A.M.); Scott_Butler@urmc.rochester.edu (J.S.B.); 3 Department of Pathology and Laboratory Medicine, Emory University School of Medicine, Atlanta, GA 30322, USA; E-Mail: mohammad.a.alam@emory.edu

**Keywords:** cholera, Type 3 Secretion System, VopX, *S. cerevisiae* CWI pathway

## Abstract

Most pathogenic, non-O1/non-O139 serogroup *Vibrio cholerae* strains cause diarrheal disease in the absence of cholera toxin. Instead, many use Type 3 Secretion System (T3SS) mediated mechanisms to disrupt host cell homeostasis. We identified a T3SS effector protein, VopX, which is translocated into mammalian cells during *in vitro* co-culture. In a *S. cerevisiae* model system, we found that expression of VopX resulted in a severe growth defect that was partially suppressed by a deletion of *RLM1*, encoding the terminal transcriptional regulator of the Cell Wall Integrity MAP kinase (CWI) regulated pathway. Growth of yeast cells in the presence of sorbitol also suppressed the defect, supporting a role for VopX in destabilizing the cell wall. Expression of VopX activated expression of β-galactosidase from an RLM1-reponsive element reporter fusion, but failed to do so in cells lacking MAP kinases upstream of Rlm1. The results suggest that VopX inhibits cell growth by stimulating the CWI pathway through Rlm1. Rlm1 is an ortholog of mammalian MEF2 transcription factors that are proposed to regulate cell differentiation, proliferation, and apoptosis. The collective findings suggest that VopX contributes to disease by activating MAP kinase cascades that elicit changes in cellular transcriptional programs.

## 1. Introduction

Of the more than 250 different serogroup strains of *V. cholerae*, only two, O1 and O139, cause epidemic cholera [1,2]. Epidemic strains use the toxin co-regulated pilus for colonization and cholera toxin to elicit the watery diarrhea that is the hallmark of the disease. Although strains belonging to other serogroups may also encode TCP (the toxin co-regulated pilus) and/or CT (cholera toxin), such non-O1/non-O139 serogroup strains more commonly carry other virulence factors [3,4,5,6,7,8]. For example, ~30%–40% of pathogenic non-O1/non-O139 serogroup strains carry a Type 3 Secretion System, which has been shown to be essential for colonization and disease in murine and rabbit models of infection [9,10,11,12].

Although bacterial Type 3 Secretion Systems share a conserved mechanism for delivering proteins into the cytosol of eukaryotic host cells, the translocated proteins (called effectors) are not conserved at the amino acid sequence level. Using a β-lactamase-based reporter system, we identified translocated proteins within the T3SS genomic island of O39 serogroup strain AM-19226 [13,14]. One of the translocated proteins, named VopX, is predicted to include 252 amino acids adopting a structure composed largely of alpha-helices [13]. Although VopX is conserved among T3SS-positive *V. cholerae* strains classified in the alpha-clade, it is otherwise unique in that no homologs are found in other T3SS-positive pathogens. Furthermore, VopX has no sequence similarity to proteins of known function. Thus, uncovering the activities of novel effector proteins such as VopX presents experimental challenges.

One approach to study effector protein function has been to use *S. cerevisiae* as a model system [15,16,17,18,19,20,21]. Four MAP-Kinase (MAPK) signaling pathways have been described for *S. cerevisiae*, and many include proteins that are well conserved in mammalian cells [22,23]. We previously screened yeast strains deleted for different components of MAPK cascades, and found that deletion of *RLM1*, the terminal transcriptional regulator of the Cell Wall Integrity (CWI) pathway, suppresses VopX toxicity [13]. The CWI pathway serves to regulate cellular responses to different stressors that damage or perturb the cell wall [24]. Signals relayed through the MAPK cascade result in the expression of transcription factors responsible for synthesis of cell wall components and actin organization. Additional lines of evidence indicate that the CWI pathway can also respond to hypoosmolarity, pH insults and heat shock, and data support a role for cross talk between the High Osmolarity Glycerol (HOG) and CWI pathways in response to cellular stresses [25,26,27].

Our previous studies did not examine whether VopX directly affects the activity of Rlm1 or results in activation of Rlm1 responsive genes. Accordingly, we further investigated the VopX-induced growth defect and interactions with components of the CWI pathway. Using a transcriptional reporter to measure the ability of Rlm to activate a known promoter element and RT-PCR to determine whether Rlm1 target genes respond to VopX expression, we demonstrate that VopX activity results in a cellular response that signals through the CWI MAPK pathway. The response requires protein kinases acting upstream of Rlm1, suggesting that VopX interacts at the top of the MAPK cascade. In addition to the growth defect phenotype, we present evidence suggesting that VopX activity disrupts the actin organization and cell cycle of *S. cerevisiae*. Our combined results support a hypothesis for VopX interaction with major signaling components that feed though the CWI pathway to result in pleiotropic phenotypes.

## 2. Results and Discussion

### 2.1. Full Length VopX Sequences are Required for Growth Inhibition in Yeast

**Figure 1:**
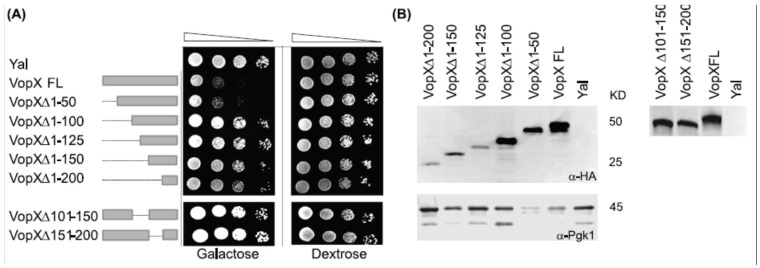
Deletion analysis identifies sequences important for function. (**A**) VopX deletions beyond the *N*-terminal 50 amino acids attenuate yeast inhibition. Strains were grown overnight at 30 °C in Synthetic Complete Dextrose (SCD) media to repress VopX expression, then 10-fold serially diluted (10^−1^ to 10^−4^, represented by the triangle) and spotted on media containing either dextrose to repress VopX expression, or galactose to induce VopX expression. VopX FL represents the predicted full length VopX protein, consisting of 252 amino acids. The doublet likely represents degradation or cleavage of a *C*-terminal protein fusion tag that is part of the expression construct; (**B**) Western analysis identified full length and deletion mutant VopX proteins, using an anti-HA antibody to detect a *C*-terminal tag present in the parent vector pBG1805-VopX and derived constructs [13]. Anti-Pgk1 antibody was used as a loading control.

To determine whether VopX activity is localized to a particular domain, we constructed a nested set of serial 50 amino acid deletions in the VopX coding sequence using pBG1805-VopX as the parent construct [13]. Deletion constructs were used to transform *S. cerevisiae* strain BY4742, and the resulting strains were grown under non-inducing conditions, 10-fold serially diluted, and plated on media containing 2% galactose to induce *vopX* expression. All strains grew equally well under non-inducing conditions (dextrose, Figure 1A). As previously shown, the strain carrying pBG1805-Yal, which encodes a random sequence used as a negative control, grew at the highest dilution (10^−4^), whereas pBG1805-VopX showed 10–100-fold growth inhibition [13,28]. Deleting the first 50 amino acids of VopX did not alter the phenotype, but deleting the first 100 amino acids and any additional amino acids relieved growth inhibition. Similarly, a protein with amino acids 101–150 or 151–200 deleted was not toxic when expressed in yeast.

As predicted by analogy with effector proteins from other species, the *N*-terminal 50 amino acids likely encode sequences required for protein translocation, and may be dispensable for activity [14]. Although deleting any other 50 amino acid segment perturbs protein function, the resulting mutant protein can be detected by Western analyses using an anti-HA antibody, suggesting that the deletions still produce a stable protein (Figure 1B). It is therefore likely that VopX activity requires the near-full length protein.

### 2.2. Temperature and Sorbitol Alter the VopX Induced Growth Defect

We rationalized that if Rlm1 is a putative target of VopX activity or if VopX interacts with components of the CWI pathway, then the VopX-induced growth defect may be responsive to changes in growth temperature or the presence of sorbitol. Temperature can affect general cellular metabolism as well as the composition and structural properties of the cell wall. The addition of sorbitol to growth media results in a hypotonic environment, which stabilizes the cell wall under conditions of osmotic stress [22]. We predicted that sorbitol would therefore alleviate the growth defect if VopX activity produced a similar stress state.

**Figure 2:**
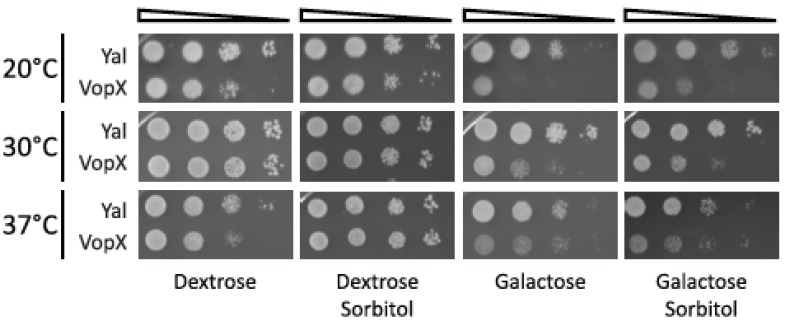
The VopX growth defect is temperature sensitive and can be partially relieved by sorbitol at 20 °C. 10-fold serial dilutions of cultures grown at 30 °C were spotted on plates containing either dextrose (represses *GAL* promoter) or galactose (induces *GAL* promoter) to control VopX and Yal expression, and with or without 1M sorbitol. Plates were incubated for 72 h at the temperatures indicated.

After growth under non-inducing conditions, strains expressing the wild-type VopX protein or the random control sequences (Yal) were serially diluted and plated on media containing either galactose alone, or galactose and 1M sorbitol, and incubated at 20 °C, 30 °C or 37 °C. The results are shown in Figure 2. We again observed a 10–100-fold growth defect when plates were incubated at 30 °C, which was not altered in the presence of sorbitol. Interestingly, we did not observe toxicity in the presence or absence of sorbitol when strains were incubated at 37 °C, suggesting that VopX activity or protein stability is temperature sensitive or altered by the cells’ heat shock response. However, the growth defect was enhanced to 100–1000 fold at 20 °C. The phenotype could be partially alleviated by sorbitol, suggesting that osmotic stress at lower temperatures increased VopX associated toxicity.

### 2.3. VopX Induces Rlm1 Responsive Element-Promoter Activity

There are two simple interpretations for the ability of an *RLM1* deletion strain to suppress the VopX induced growth defect. VopX could directly interact with Rlm1, or could initiate signaling via interactions higher in the MAPK hierarchy, which would ultimately result in an active Rlm1 protein. Five proteins function upstream to activate Rlm1 in the CWI signaling pathway: Rho1, Pkc1, Bck1, Mkk1/2, and Slt2. *BCK1* or *SLT2* deletions failed to suppress the growth defect caused by VopX expression, consistent with a report that overstimulation at a point upstream of Bck1p negatively effects growth of *BCK1* or *SLT2* mutants [29]. Thus, if VopX stimulates the pathway above the MAP-kinases and affects branching pathways related to the actin cytoskeleton, *BCK1* and *SLT2* deletions might fail to restore growth.

**Figure 3:**
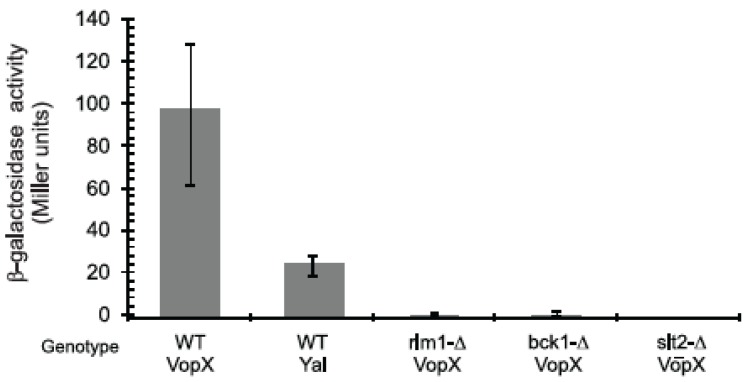
VopX expression activates Rlm1 responsive promoter element activity. A plasmid based PRM5-*lacZ* reporter fusion was introduced into *S. cerevisiae* strains possessing an intact CWI pathway or deleted for different MAPK components. Strains expressing VopX or Yal control sequences were grown at 30 °C under conditions promoting VopX or Yal expression, then processed for the assay. Three colonies were used for each strain, and the assay was repeated with similar results. Not shown: WT strains grown under non-inducing conditions expressed 13 Miller units of activity when carrying Yal sequences and 15 Miller units of activity when carrying VopX.

We therefore tested whether expression of VopX causes activation of an Rlm1-dependent promoter. Using a *lacZ* transcriptional reporter fusion to sequences encoding the *RLM1*-responsive promoter element of *PRM5*, we measured Rlm1 activity when strains were expressing either VopX or Yal (Figure 3, [30]). Expression of VopX in wild type cells causes a five-fold increase in expression of β-galactosidase activity compared to the negative control Yal (Figure 3). In contrast, VopX caused little or no activation of the promoter fusion in cells with deletions of *RLM1*, *BCK1* or *SLT2*. The findings confirm that VopX expression causes activation of the *RLM1*-responsive promoter element in an Rlm1 dependent manner. We therefore conclude that VopX activity likely targets components in the CWI pathway upstream of Rlm1, causing pleiotropic phenotypes that include altered Rlm1 target gene expression. Importantly, however, Rlm1 associated gene activation that responds to VopX expression requires the activity of the upstream MAP kinases Bck1 and Slt2. Consistent with this observation, immunoprecipitaiton of epitope-tagged VopX in yeast, followed by mass spectrometry analysis, did not identify interactions with Rlm1 (data not shown). We therefore propose that VopX most likely interacts with upstream CWI pathway protein(s) rather than directly with Rlm1, resulting in pleiotropic phenotypes related to cytoskeletal architecture and cell cycle control.

### 2.4. VopX Expression Activates Expression of Genes Downstream of RLM1

**Figure 4:**
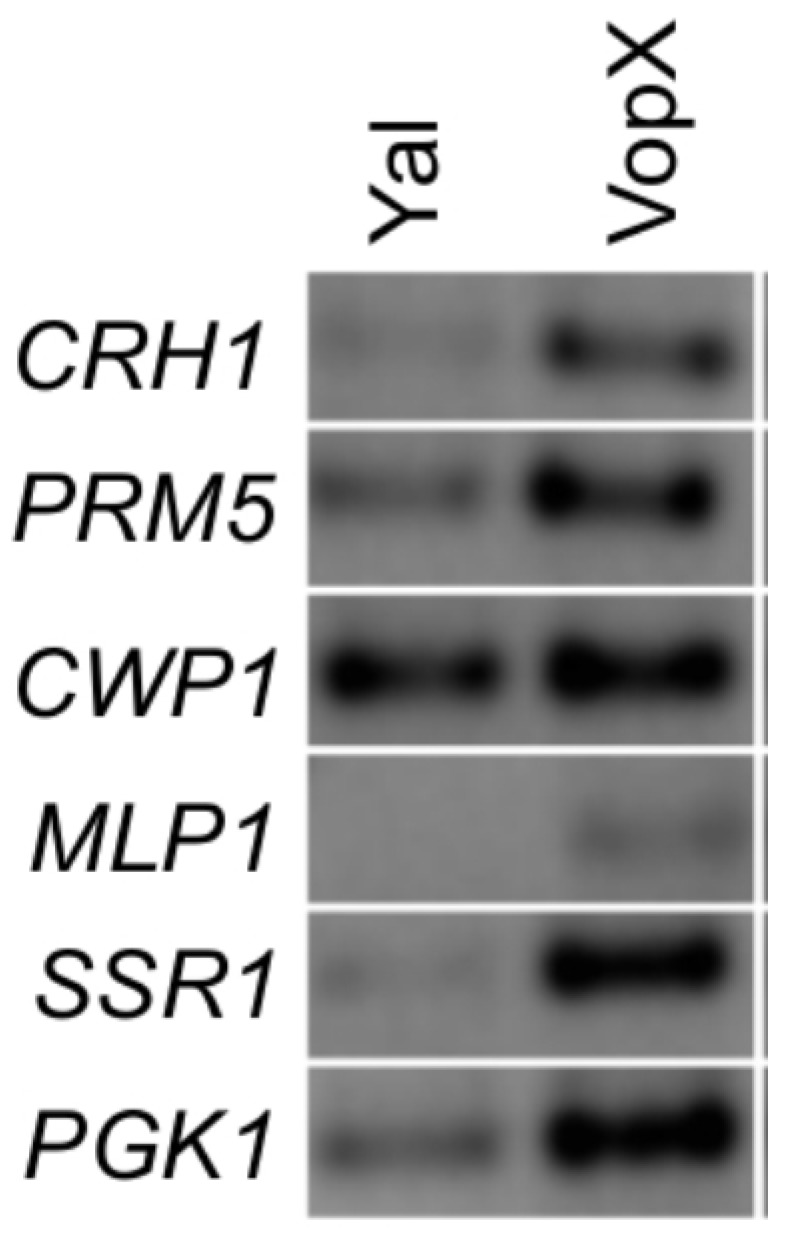
VopX expression influences the expression of Rlm1 responsive genes. Semi-quantitative RT-PCR was performed on RNA extracted from cultures grown in the presence of galactose. Primers were designed to amplify genes that are targets of Rlm1 transcriptional activation, encoding cell wall proteins, an Rlm1 associated protein, or stress response proteins.

The targets of Rlm1 activity include genes encoding cell wall proteins (e.g., *CWP1* and *SSR1*), *MLP1*, whose protein product associates with Rlm1, and others identified as induced by cell wall stress (*CRH1* and *PRM5*) [31]. Because the results of Figure 3 indicate that VopX expression can result in Rlm1 transcriptional activity and subsequent expression from downstream target promoters, we performed semi-quantitative RT-PCR to determine whether other known targets of Rlm1 activity respond to VopX. As shown in Figure 4, we observed increased expression of *CRH1*, *PRM5*, *MLP1* and *SSR1* in strains expressing VopX compared to strains expressing the Yal control sequences. *PGK1* (encoding an enzyme involved in gluconeogenesis) was reported to be negatively regulated by Rlm1, however, we observed an increase in expression that was correlated with VopX [31]. We did not observe an increase in *CWP1* expression, which could be explained by the fact that *CWP1* is not controlled exclusively by Rlm1 [31]. The results therefore provide additional supporting evidence that VopX induces MAPK signaling through the CWI pathway, resulting in an altered transcriptional profile that is at least in part mediated by Rlm1.

### 2.5. VopX Expression Alters Cell Cycle Progression

**Figure 5:**
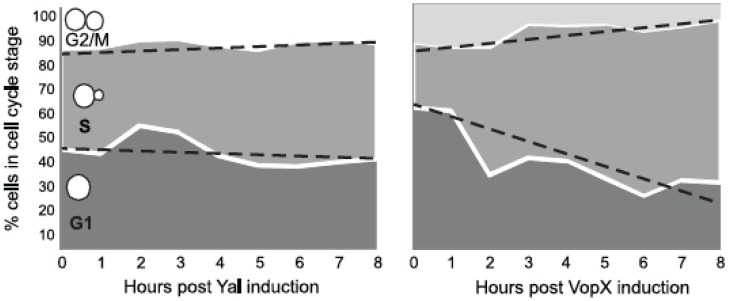
VopX expression alters yeast cell cycle progression. *S. cerevisiae* strains carrying pBG1805-VopX or pBG1805-Yal were grown to log phase in repressing medium (2% SCD-URA), normalized by OD_600_, washed, and resuspended in inducing medium (2% SCG-URA). Samples of each strain were removed every hour for eight hours and imaged by light microscopy. For each strain, ~200 randomly selected yeast cells were counted at each time point and growth phases recorded.

VopX-induced toxicity could result from growth arrest at a specific stage of the cell cycle or more randomly from inhibition at different checkpoints. Previous studies established that visual observations of an asynchronous population can reveal cell cycle delays, since the number of cells observed in each stage of the cell cycle is correlated with the amount of time the population is in that stage [24]. We therefore performed standard visual cell cycle stage analysis using bud emergence as a marker for entry into S phase and large bud size as a marker for the G2/M transition, inspecting cells every hour for eight hours after inducing VopX expression. Figure 5 summarizes the data for strains expressing either the control sequences (Yal) or VopX. While the percentage of cells in G1 (no bud), S and G2/M phases remained relatively constant over the course of eight hours in the control strain, the percentage of cells in S phase increased when VopX was expressed. We recorded a corresponding decrease in the number of cells in G1 and G2/M phases, suggesting that expression of VopX resulted in either a slowing of progression through S phase or a halt at the corresponding checkpoint prior to the G2/M transition. The accumulation of cells with small buds mirrors that seen in cell wall integrity checkpoint arrested cells [32,33].

### 2.6. VopX Disrupts Actin Localization during the Budding Stage of the Cell Cycle

**Figure 6:**
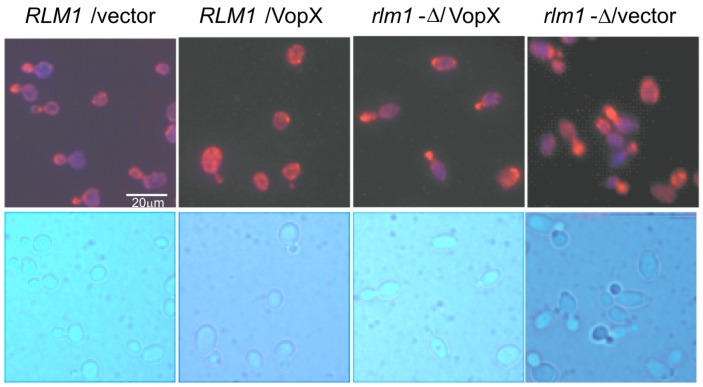
Expression of VopX depolarizes actin cytoskeleton. Wild-type and *rlm1*Δ strains carrying empty vector (pBG1805) or plasmid containing VopX were grown in the presence of galactose for 5 h. Cells were fixed, processed and stained with Alexa Fluor 546 phalloidin to visualize the actin cytoskeleton.

The actin cytoskeleton is an important component for yeast cell wall maintenance, and the CWI kinase cascade is linked to actin polarization [34]. Having shown that VopX expression results in CWI pathway activation via Rlm1, we investigated whether VopX expression also disrupts actin dynamics. We used Alexa Fluor 546 conjugated phalloidin to stain cells from strains expressing VopX or Yal, in wild-type and *RLM1* deletion backgrounds. As expected in wild-type cells, actin is localized to the budding pole of dividing cells (Figure 6). In contrast, cells expressing VopX appear to have actin more evenly distributed even though bright field images can identify budding cells. Importantly, actin polarization to cell buds is restored in *RLM1* deletion cells expressing VopX. These results support our conclusion that VopX influences CWI signaling activity and actin depolarization through activation of Rlm1. Moreover, the actin defects and cell cycle phenotypes suggest that VopX interferes with cell wall remodeling and cytoskeletal dynamics required for bud enlargement and completion of the cell wall integrity checkpoint [32,33].

## 3. Experimental Section

### 3.1. Strains, Media, and Standard Techniques

Yeast strain BY4742 (MATα *his3-*Δ*1 leu2-*Δ*0 lys2-*Δ*0 ura3-*Δ*0*) was used as the parental yeast strain for all studies. DH5α (F^−^ (ϕ80*dlac*ΔM15) (*lacZYA-argF* Δ*U169*) *endA1 recA1 hsdR17 deoR thi-1 supE44gyrA96* (Nalr) *relA1*) was used as the standard *E. coli* cloning strain. Ampicillin was used at 100 µg/mL. Yeast strains were grown in Synthetic Complete (SC) media supplemented with dextrose (SCD) to a final concentration of 2% or galactose (SCG) to a final concentration of 2% or 1% each galactose/raffinose. Molecular biology procedures were performed according to standard protocols [35]. Primer sequences are available upon request.

### 3.2. Yeast Growth Inhibition Assay

Yeast strains were grown in SCD-Ura medium (SCD medium lacking uracil and also leucine when required) at 30 °C on a roller drum to an optical density at 600 nm (OD600) of 1 to 1.5. After adjusting the OD600 to 1.0, 10-fold serial dilutions to 10^−5^ were made, and 7 µL of each of the dilutions was spotted onto SCD-Ura-Leu, or SCG lacking uracil and leucine when required (SCGal-Ura-Leu). The plates were incubated at 30 °C and photographed at 72 h (day 3). All yeast deletion mutations were verified by PCR analysis using gene-specific primers.

### 3.3. pCHS02 Plasmid Construction

The URA marker in plasmid PRM5-*lacZ* was replaced with a LEU marker to allow for selection of pBG1805 based plasmids and the PRM5-*lacZ* plasmid based reporter [30]. PRM5*-lacZ* was digested with SmaI and SbfI, and the linear fragment carrying vector and transcriptional reporter fusion sequences was gel purified. The LEU marker in YCplac111 was amplified by PCR using gene specific primers that introduced SmaI and SbfI sites to facilitate cloning. The resulting plasmid, pCHS02, was verified by restriction analysis, PCR, and functional tests for auxotrophic complementation [30].

### 3.4. β-Galactosidase Assay

Single colonies were inoculated into 5 mL of 2% raffinose synthetic complete media lacking uracil and leucine (SCR-URA-LEU) and grown with aeration at 30 °C for 24 to 72 h to mid log phase (OD600: 0.2–0.5). Cultures were then divided to two tubes, with one tube induced for VopX or Yal production with 2% galactose, and one remaining as an uninduced control. Both induced and uninduced strains were grown for 2 h at 30 °C. Beta-galactosidase assays were then performed on each sample, as previously described [36]. Briefly, approximately 1 × 10^6^ cells were centrifuged at 13.2 k RPM for 3 min, and resuspended in 1 mL Z Buffer [35]. 20 µL chloroform, and 10 µL 0.1% SDS were added and each sample was vortexed for 15 s to lyse the cells. Samples were incubated 15 m at 30 °C. 25 µL of suspension was added to a 96 well plate, followed by 175 µL of Z buffer without BME for a final volume of 200 µL. To initiate the reaction, 50 µL ONPG solution (10 mg/mL in Z-Buffer without BME) was then added to each sample and the plate was immediately measured at 420 nm at 5 m intervals for one hour in a PowerWave XS spectrophotometer (Bio-Tek, Winooski, VT, USA). The unit for this kinetic assay is equal to micromoles of ONPG hydrolyzed per minute per OD600 unit.

### 3.5. RT-PCR

Single colonies were inoculated into 3 mL of SCD media lacking uracil and leucine and grown with aeration at 30 °C for 16 to 18 h to late-log phase. Each sample was then subcultured 1:100 into SCG-URA and grown to mid log phase (OD600: 0.5–0.8). Cells were pelleted and snap-frozen at −80 °C. RNA was collected by Trizol/phenol-chloroform extraction and purified according to manufacturer’s instructions and standard protocols [35]. Semi-quantitative RT-PCR was performed with Superscript III for 35 cycles and visualized by gel electrophoresis (ThermoFisher Scientific, Waltham, MA, USA).

### 3.6. Actin Staining

Yeast strains were grown overnight in SC-Ura + raffinose media at 30 °C. Fresh SC-Ura + raffinose media were inoculated with the overnight culture and allowed to grow for 4 h. Galactose was added to the cultures and grown for another 5 h at 30 °C. Cells were fixed by adding formaldehyde directly to the culture media to a final concentration of 3.7% and incubating for 20 min at room temperature with gentle shaking. Cells were harvested; pellets were resuspended in 3.7% formaldehyde solution in PBS, and incubated at room temperature for 30 min with gentle agitation. Cells were then washed with PBS three times and resuspended in 0.1% Triton X-100 in PBS. After washing three times with PBS, yeast cells were resuspended in 1 µM Alexa Fluor 546 phalloidin (ThermoFisher Scientific, Waltham, MA, USA) and incubated for 30 min at 4 °C with slow rocking protected from light. Cells were washed with PBS, resuspended in mounting media and visualized using an Olympus BX41 microscope (Olympus America, Inc., Center Valley, PA, USA) with the 100x objective and QCapture software (Surrey, BC, Canada).

## 4. Conclusions

We previously identified Rlm1, the terminal transcription factor of the CWI signaling pathway in *S. cerevisiae*, as a putative target of VopX activity. Subsequent studies presented here demonstrate that VopX expression can indeed induce signaling through Rlm1, resulting in growth inhibition and the activation of Rlm1 responsive promoters. However, the results of transcriptional reporter fusion assays indicate that MAP kinases upstream of Rlm1, which control Rlm1 activation, are required for the VopX induced phenotype. We therefore predicted that VopX activity may intersect with the CWI pathway upstream of Rlm1, and examined other phenotypes associated with CWI pathway functions. In support of our prediction, we found that VopX expression induced alterations in actin cytoskeleton organization during cell division and resulted in cell cycle arrest. The collective phenotypes induced by VopX expression therefore suggest an interaction with proteins that function early in the CWI cascade, perhaps at the level of Rho1 or Pkc1. Further studies will examine the effect of VopX expression in mammalian cells, targeting homologous proteins whose disrupted function would likely contribute to disease caused by infection with T3SS-positive *V. cholerae* expressing VopX.
